# Clinical outcomes of patients with multivessel coronary artery disease treated with robot-assisted coronary artery bypass graft surgery versus one-stage percutaneous coronary intervention using drug-eluting stents

**DOI:** 10.1097/MD.0000000000017202

**Published:** 2019-09-20

**Authors:** Chieh-Shou Su, Ching-Hui Shen, Keng-Hao Chang, Chih-Hung Lai, Tsun-Jui Liu, Kuan-Ju Chen, Tzu-Hsiang Lin, Yu-Wei Chen, Wen-Lieng Lee

**Affiliations:** a Cardiovascular Center, Taichung Veterans General Hospital, Taichung; b Institute of Clinical Medicine, and Department of Medicine, National Yang-Ming University School of Medicine, Taipei; c Department of Anesthesiology, Taichung Veterans General Hospital, Taichung; d School of Medicine, National Yang-Ming University, Taipei; e Department of Internal Medicine, Cheng Ching Hospital, Taichung; f Department of Medicine, National Yang-Ming University School of Medicine, Taipei; g Department of Emergency Medicine, Taichung Veterans General Hospital, Taichung, Taiwan.

**Keywords:** multivessel coronary artery disease, percutaneous coronary intervention, robot-assisted coronary artery bypass graft surgery

## Abstract

A number of studies have reported on treatment outcomes of coronary stenting (PCI) for multivessel coronary artery diseases (MVD), and compared them with the conventional coronary artery bypass grafting (CABG). However, the clinical outcomes of robot-assisted CABG (R-CABG) in comparison with PCI in MVD patients have not been investigated.

We recruited retrospectively MVD patients receiving R-CABG and PCI with drug-eluting stents for all vessels in one stage between January 2005 and December 2013 at our institution with at least 3 years of outcomes were retrospectively recruited and analyzed.

A total of 638 MVD patients were studied. Among them, 281 received R-CABG, and 357 received PCI. Similar complete revascularizations were achieved in both groups (R-CABG: 40.2%, PCI: 41.5%, *P* = .751). The residual stenosis was 4.1 ± 4.4 in the R-CABG group, and comparably 3.5 ± 3.7 in the PCI group (*P* = .077). Patients in the R-CABG group were younger, with more severe coronary artery disease (CAD) and had more background risk factors. The in-hospital and long-term mortalities as well as the incidence of TLR, myocardial infarction (MI), stroke were all similar between groups. But the incidence of TVR and any revascularization were lower in the R-CABG group. The long-term mortality was predicted by age, left ventricular ejection fraction, and chronic kidney disease, but not by the revascularization modality, completeness of revascularization, nor residual SYNTAX scores. The last 3 factors were not predictors of long-term TLR, TVR, MI, and stroke.

The in-hospital and long-term survival rates of MVD were similar for both the R-CABG and PCI groups. But the R-CABG group had rates of TVR and any revascularization lower than PCI. Revascularization modality, completeness of revascularization, and residual SYNTAX scores were not predictors of in-hospital and long-term mortalities, MI, and stroke in real-world practice. R-CABG was associated with lower rates of TLR and TVR, and is likely a safe and effective treatment and an alternative choice of PCI for MVD patients who have low surgical risks.

## 1 Introduction

Patients with multivessel coronary artery disease (MVD), compared single-vessel coronary artery disease (CAD), have more comorbidities, cardiovascular risks, and higher prevalence of left ventricular dysfunction. Both coronary artery bypass surgery (CABG) and percutaneous coronary intervention (PCI) are established as standard treatments for MVD and are considered equally safe and effective. It remains debatable as to whether other modality may be an even more effective treatment.^[1–4]^ Recently, the robot-assisted CABG (R-CABG) has been applied more widely to treat complex CAD,^[5–8]^ valvular heart disease (VHD),^[9,10]^ and congenital heart disease^[11,12]^ mainly because its advantages over the conventional open heart surgery, namely: shorter ICU and hospital stays, lower blood transfusion requirement, fewer post-operative complications, and better post-operative quality of life. R-CABG combines the dual benefits of PCI and the conventional CABG (C-CABG). It has proven efficacy and effectiveness in treating complex CAD. Its popularity is reflected by its increasing operation volume worldwide. In our previous study, we showed that R-CABG is feasible for patients with stable LM disease and high SYNTAX scores, and it is an effective alternative to C-CABG in treating LM disease patients with fewer risk factors. Revascularization modality per se is not a determinant for long-term mortality in our real-world practice.^[7]^ However, the clinical outcome of R-CABG versus PCI with drug-eluting stents (DES) for multivessel CAD has not been reported. Here, we retrospectively analyzed MVD patients receiving R-CABG or PCI with DES, and compared the two treatment modalities in terms of their clinical data, and outcomes.

## 2 Methods and materials

We retrospectively analyzed patients, with angiographically proven MVD, who had received R-CABG or PCI with DES for all vessels in one stage at our institute between January 2005 and December 2013. We excluded patients with cardiogenic shock, cardiac arrest, end-stage renal disease, or prior history of CABG, and also those who had received PCI without DES implantation, or PCI done in >1 stages, had hybrid therapy of CABG and PCI, underwent CABG combined with other open heart surgeries, or received a medical therapy instead of CABG or PCI. MVD was defined, according to angiographic findings, as severe stenosis (≥70%) by the presence of in at least 2 diseased major epicardial coronary arteries. The choice of revascularization modality was primarily determined by the current guidelines, and also at the discretion of the attending physicians. As a general rule, patients with proven MVD with high SYNTAX scores (≥ 33) were recommended to receive CABG as the first choice of therapy and PCI as an alternative therapy on the condition that they had declined CABG or had high surgical risks. Those with low SYNTAX scores (≤ 22) were recommended to receive PCI as the first treatment strategy, whereas those with intermediate SYNTAX scores (≥22 and <33) were recommended to receive either CABG or PCI. Patients who opted for surgical revascularization received either conventional-CABG (C-CABG) or R-CABG, depending on the co-morbidities, frailty, personal willingness, and financial capability. Only those who received R-CABG were enrolled in this study. Both PCI and R-CABG were carried out according to the standard practice of our institute. The procedures of both revascularization approaches are described briefly as follows. R-CABG was performed with Da Vinci robotic system under generalized anesthesia through three pencil-sized incisions along the left anterior axillary line over the 2nd, 4th, and 6th intercostal spaces. First, the cardiovascular surgeon harvested the left radial artery and the left internal mammary artery (LIMA) graft inside the chest through an endoscope, and then performed a pericardiotomy to expose the native coronary arteries. An incision about 2.5 to 3 cm long was made over the 2nd intercostal space adjacent to the sternal bone for creating the anastomosis for the harvested radial artery to the LIMA graft in an end-to-side manner. Next, an off-pump hand-sewn LIMA-LAD anastomosis was performed again in an end-to-side manner and LIMA-radial artery-sequential grafts were anastomosed to the diagonal artery, left circumflex artery, or posterior descending artery, depending on which arteries were involved, via an 8-cm left anterolateral thoracotomy. The percutaneous MVD interventions were performed by experienced interventionalists according to the standard practice of our institute. The choice of using the DES or bare-metal stent (BMS) was made by the operators based on lesion characteristics and the financial capability of the patient. PCI for MVD may be carried out in stages and using non-DES, an approach which would have complicated data collection and analysis. For this reason, only patients treated with DES for all vessels in a single stage were studied.

Baseline demographics were retrospectively extracted from medical records of the hospital database. In-hospital outcomes were carefully retrieved by reviewing the medical records and charts. Long-term outcomes in the post-hospitalized periods were collected from the notes of outpatient department recorded in the hospital database or in the case they were followed up at other hospitals during the study period through phone calls to patients made by the researcher assistant or nurse. The study protocol was reviewed and approved by the Institutional Review Board/Ethics Committee of Taichung Veterans General Hospital, Taichung, Taiwan.

### 2.1 Statistical analysis

Continuous variables were presented as mean ± SD, and categorical variable as frequencies and percentages. Differences in continuous variables were analyzed using the Mann–Whitney *U* test. Categorical variables were analyzed using the chi-square test. Logistic regression analysis was performed to determine the independent predictors for in-hospital and long-term follow-up mortalities, and other outcomes. Variables with a *P* value of <.10 in the univariate analysis were included for the multivariate analysis. Between-group differences were considered statistically significant if *P* value was <.05. We used the SPSS 19.0 (SPSS Inc., Chicago, IL) statistical software for all out analyses.

## 3 Results

### 3.1 Baseline characteristics of all patients with MVD

A total of 638 patients were recruited for this study. Among them, 281 received R-CABG, and 357 received PCI (corresponding to 4.9% of all PCI procedures at our institute during the study period). Their baseline characteristics are shown in Table 1. Patients in the R-CABG group, compared with the PCI group, were younger, with higher serum levels of creatinine, higher SYNTAX scores, and higher prevalences of hypertension, dyslipidemia, and cigarette smoking. At the same time, R-CABG patients also had fewer incidences of prior myocardial infarction (MI), acute coronary syndrome (ACS), as well as lower hemoglobin concentrations and lower Euro scores. The left ventricular ejection fraction (LVEF) was similar between the 2 groups. After revascularization by CABG or PCI, the incidences of residual stenosis appeared lower in R-CABG group, but this difference was not statistically significant (4.1 ± 4.4 vs 3.5 ± 3.7, *P* = .077). SYNTAX revascularization index (SRI) was also higher in the R-CABG group (89.9% ± 9.2% vs 87.5% ± 11.4%, *P* = .001). Proportions of patients with complete revascularization were similar across the two groups (40.2% vs 41.5%, *P* = .751).

**Table 1:**
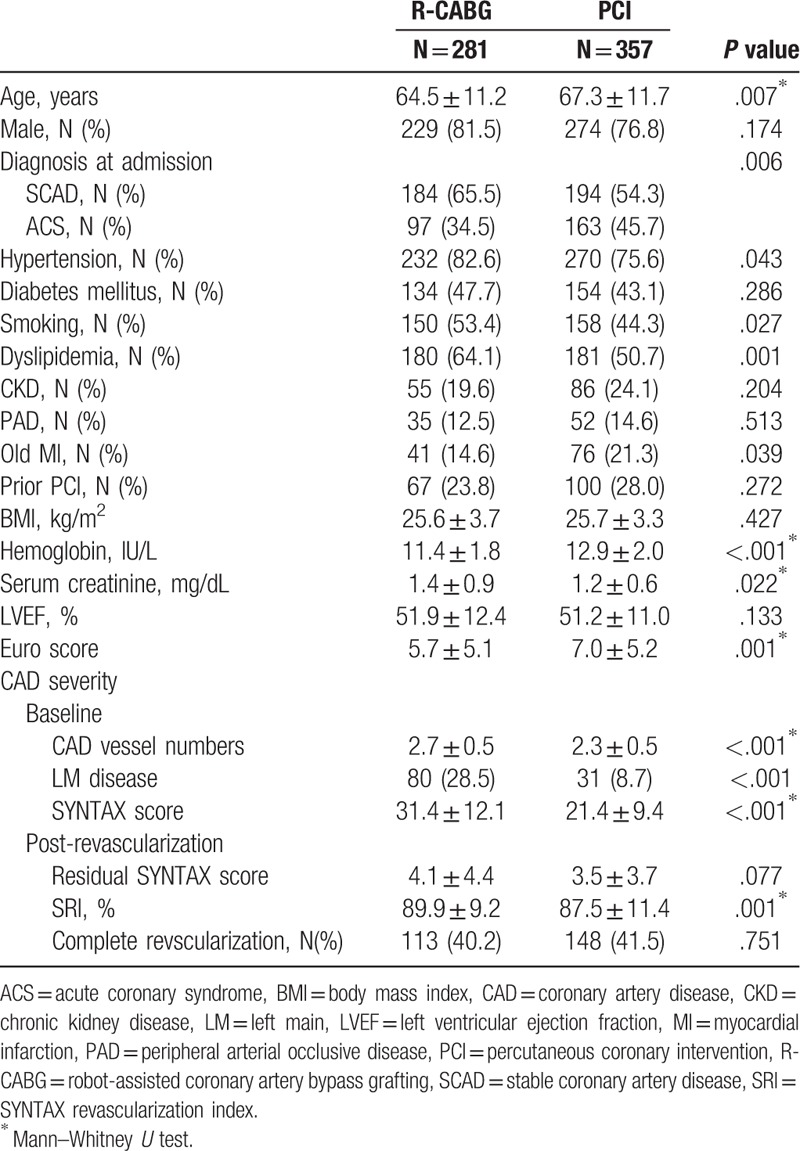
Demographic characteristics of coronary multiple vessel disease patients who underwent R-CABG versus PCI.

### 3.2 In- and post-hospital clinical outcomes

The in- and post-hospital clinical outcomes are shown in Table 2. Patients in the R-CABG group required more assistance with intra-aortic balloon pump (IABP) and extracorporeal membrane oxygenation (ECMO). The lengths of ICU and total hospital stays were also longer in the R-CABG group. Despite of this, no difference was found in terms of in-hospital mortality between groups.

**Table 2:**
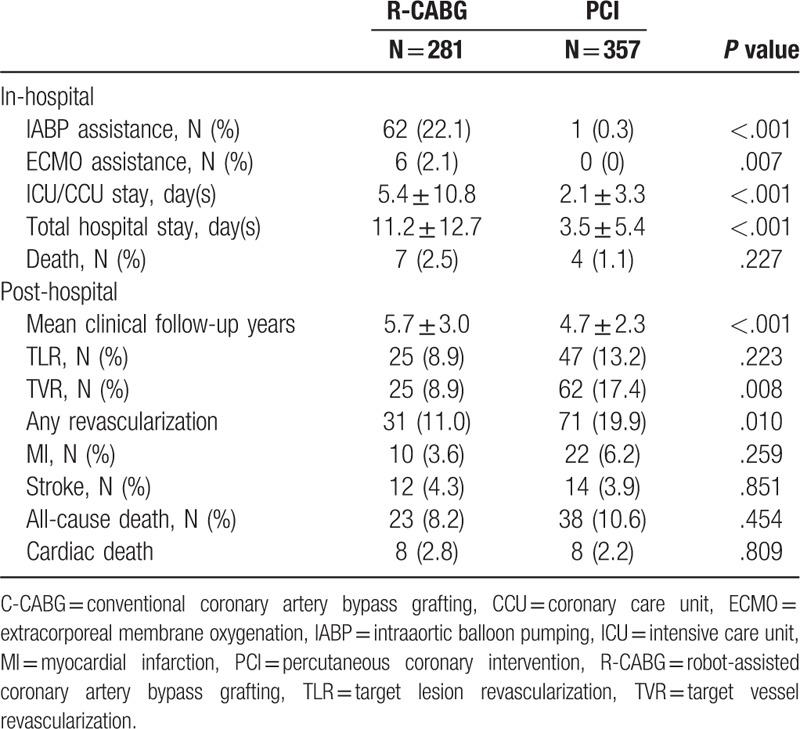
In- and post-hospital clinical outcomes of multiple vessel coronary disease patients who underwent R-CABG versus PCI.

After hospital discharge, R-CABG patients were followed up for 5.7 ± 3.0 years, which was longer than 4.7 ± 2.3 years in the PCI group (<*P* = .001). The incidences of TLR, MI, stroke, any cause of mortality, and cardiac death were similar between the 2 groups, except that the incidences of TVR and any revascularization in the R-CABG group were significantly lower than in the PCI group.

### 3.3 Clinical predictors for in-hospital and long-term mortalities

The clinical predictors for in-hospital mortality are shown in Table 3. and post-hospital long-term mortality in Table 4. Logistic regression analyses showed that age, LVEF, and chronic kidney disease (CKD) were independent predictors for post-hospital long-term mortality, whereas revascularization modality, completeness of revascularization, and residual SYNTAX scores were not. Moreover, we found no single factor predicting the in-hospital mortality.

**Table 3:**
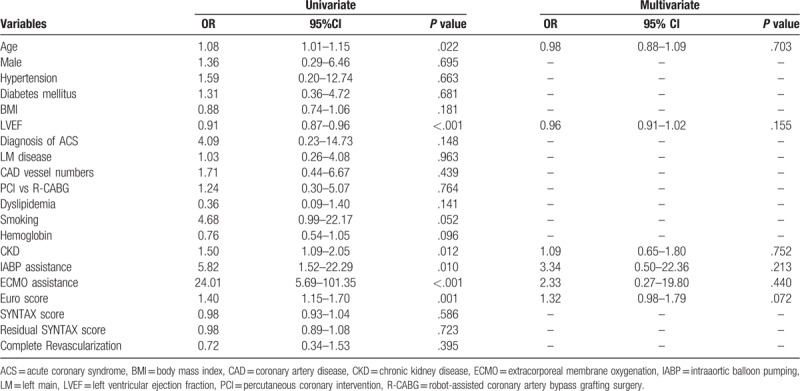
Logistic regression analysis for in-hospital mortality of all multivessel coronary disease patients.

**Table 4:**
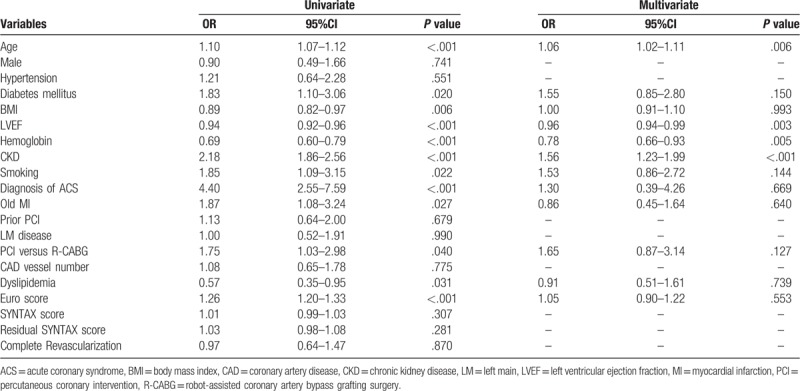
Logistic regression analysis for long-term mortality of all multivessel coronary artery disease patients.

### 3.4 Clinical predictors for major adverse cardiovascular events

The clinical predictors for major adverse cardiovascular events (such as MI, TLR, TVR, and stroke) are shown in Tables 5 to 8. Logistic regression analyses showed that age and PCI were independent predictors for TLR, while DM and PCI were independent predictors for TVR. In terms of long-term stroke, BMI was the single independent predictor. The revascularization modality, completeness of revascularization, and residual SYNTAX scores did not predict long-term TLR, TVR, MI, and stroke.

**Table 5:**
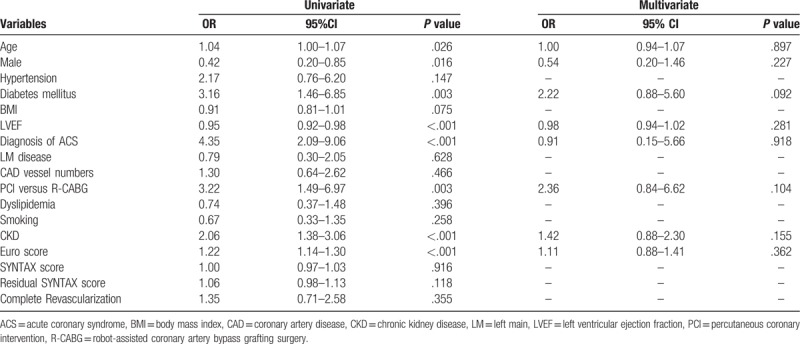
Logistic regression analysis for risk of long-term myocardial infarction in all multivessel coronary artery disease patients.

**Table 6:**
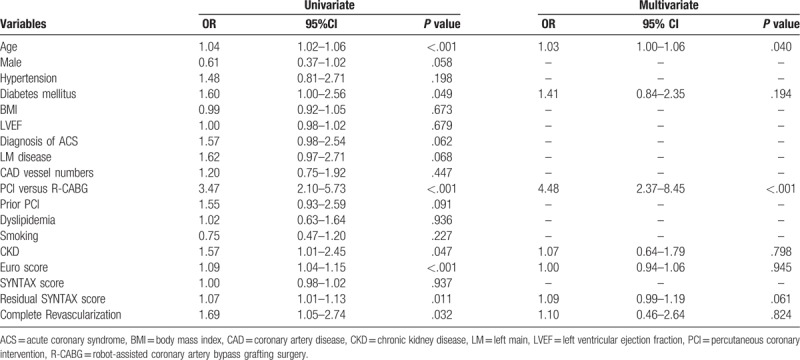
Logistic regression analysis for long-term risk of target lesion revascularization in all multivessel coronary artery disease patients.

**Table 7:**
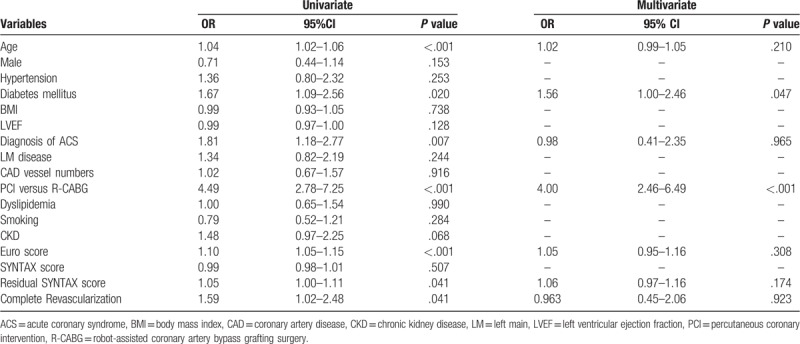
Logistic regression analysis for long-term risk of target vessel revascularization in all multivessel coronary artery disease patients.

**Table 8:**
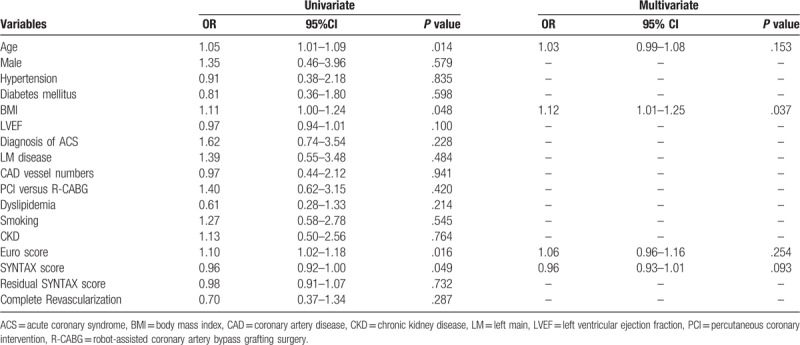
Logistic regression analysis for long-term risk of stroke in all multivessel coronary artery disease patients.

## 4 Discussion

Our major findings are the following:

(1) PCI with DES and R-CABG had equally high in-hospital and long-term survival rates as well as similar completeness of revascularization and residual SYNTAX scores. But, the PCI group had higher incidences of TVR and any revascularization;
(2) Age, LVEF, hemoglobin, and CKD (but not revascularization modality, completeness of revascularization, nor residual SYNTAX scores) were independent predictors for long-term survivals;
(3) Age and PCI were independent predictors for TLR, while DM and PCI were independent predictors for TVR; and
(4) BMI was the only independent predictor for stroke.

In the last decade or two, a number of studies on MVD patients have shown that CABG and PCI using BMS/DES yield similar clinical outcomes in terms of hard endpoints (i.e., death and MI). But CABG is more superior than PCI in terms of the need to repeat revascularizations.^[2,3]^ Our current study is the first of its kind to show differences in patient characteristics and clinical outcomes between R-CABG and PCI with DES for MVD patients in real world practice. Here, we retrospectively investigated patients with MVD receiving either R-CABG or PCI with full DES for all vessels operated in one stage. We found that both modalities had similarly low in-hospital mortality rates and high long-term survival rates, despite the PCI group had more TVR and any revascularization. Our findings suggested that R-CABG can be considered a feasible revascularization modality for MVD patients with higher lesion severity, and lower surgical risks. With the recent innovations in surgical devices and techniques, open thoracotomy surgeries have increasingly been replaced by the endoscopic approach, as the latter provides the benefits of shorter ICU and total hospital stays, lower blood transfusion requirements, better post-operative quality of life, and fewer post-operative complications. Robot-assisted surgery is proved safe and effective, and has gained popularity during the past few decades.^[13–16]^ In the field of coronary revascularization, the goal for minimally invasive approaches has led to a wider application of PCI, despite its association with more reinterventions. Over the past 2 decades, robot-assisted cardiovascular surgery using the Da Vinci system, which combines the advantages of 2 revascularization methods (like the smaller wounds, less rib retraction, less pain, prompt return to normal activities and a positive impact on the quality of life),^[5,17]^ has been increasingly used worldwide to treat congenital heart disease,^[11,12]^ VHD,^[9,10]^ and CAD.^[5–8]^ Despite the fact that contemporary standard CABG being the major approach in most hospitals across the world and its outstanding results with acceptable hospital stays and peri-operative complications,^[8]^ CABG still might not be adopted due to concerns of religions, multiple comorbidities, advanced ages and frailty and the demand of less traumatic alternatives. Fortunately, results of R-CABG are quite positive in the literature with advantages like lower hospital charges,^[18,19]^ less administration of analgesics after operation,^[20]^ shorter ICU/hospital stays and less peri-/post-operative MACCEs.^[19,21,22]^ These advantages were reported in our previous study in which R-CABG is shown feasible to treat stable LM disease patients with high SYNTAX scores and it is an effective alternative to C-CABG in LM disease patients with few risk factors.^[7]^ In our present study, revascularization modality per se was not a determinant for long-term mortality. Our patients with MVD in the R-CABG group were younger, had lower prevalence of ACS, and had a lower Euro score, but had more diseased coronary vessels, LM disease, comorbidities, and higher SYNTAX scores. In addition, in another cohort of MVD patients (N = 516) receiving R-CABG (n = 281) or C-CABG (n = 235), we found that patients in the R-CABG group were younger, and had lower CAD severity and less background risk factors. The in-hospital and long-term mortalities were lower in the R-CABG group but the incidences of TLR, TVR, MI, and stroke were not significantly different between the two groups. The long-term mortality was related to age, lower LVEF, chronic renal disease, but not residual SYNTAX scores, completeness of revascularization nor the revascularization modality (unpublished data). These phenomena essentially serve as the criteria by which attending physicians select the revascularization modality for their patients in the real-world practice, as it is essential to treat any patient as a whole, taking all of the relevant factors into account, rather than simply considering the superiority of one revascularization modality over the other.

The goals of treating MVD are to alleviate symptoms of angina and to prevent ischemic heart failure, and for the longevity of life. With growing improvements in coronary devices and techniques, PCI, despite its need for more repeated revascularization^[2,3]^ is now widely considered equivalent to CABG, with no significant differences in mid- to long-term survivals nor major adverse cardiac and cerebrovascular events (MACCE). Complete revascularization and residual SYNTAX scores after the MVD treatment are considered factors affecting long-term outcomes but their exact roles remain illusive.^[23–30]^ In our present study, both the completeness of revascularization and residual SYNTAX scores were not predictors for in-hospital nor long-term mortality, MI, TLR, TVR, and stroke. The percentage of complete revascularization (40.2% in the R-CABG group) is lower than what have been reported in the literature.^[27,30]^ This could be related to the fact that the completeness of revascularization is more difficult to achieve in R-CABG, as multiple and diffuse diseased small vessels lack an adequate graft landing zone, and anastomosing each diseased branch is technically challenging. The percentage of complete revascularization (41.5% in the PCI group) is also low compared with the reported studies.^[27,30]^ Incomplete percutaneous revascularization might be multifactorial, including lesion characteristics (e.g., totally occluded vessel, multi-site lesions, or calcified and un-dilated lesion), planned staged PCI, ischemic territory supplied by small vessels, risks of radiation overexposure, overload of contrast medium, and the issue of high costs with the multiple DES. Xu et al,^[31]^ has reported that SRI is a predictor of mid-term mortality and MACCE in patients with complex CAD who underwent PCI with DES. SRI ≧ 85% is associated with a death risk that is similar to that of complete revascularization. This might be considered a reasonable goal in complex MVD revascularization. In our present study, rates of SRI in the R-CABG and PCI groups were 89.9% ± 9.2%, and 87.5% ± 11.4%, respectively, and these high rates might explain, at least in part, why completeness of revascularization was not an independent predictor for mortality and MACCE.

In conclusion, R-CABG for MVD had similarly high in-hospital and long-term survival rates, but less TVR and any revascularization compared with PCI. Revascularization modality, completeness of revascularization, and residual SYNTAX scores were not predictors for in-hospital and long-term mortalities, MI, nor stroke. R-CABG predicted smaller likelihood of TLR and TVR and might be a safe and effective alternative to PCI for MVD patients with low surgical risks.

## 5 Study limitations

There are some limitations in our study. First, this was a retrospective, observational, and non-randomized study, and inevitably subject to limitations inherent in such experimental design. Second, the choice of revascularization modality was at the discretion of the attending physician together with the patient, a decision which was affected by financial constraints, rather than exclusively the treatment guidelines. However, we do think our study population reflected real-world practice, as the choice of revascularization modality was made to the best interests of the individual patients, who likely had taken multiple relevant factors into consideration, apart from lesion anatomy and treatment guidelines. Given the variations in co-morbidities between the 2 groups, multivariate logistic regression analysis had identified the predictors of outcomes. Third, the sample size of patients in our study was not desirably large and the PCI group came from only a minor fraction of all PCI patients we had treated in our institute. The impact of residual SYNTAX scores and incomplete revascularization on clinical outcomes likely has been underestimated. Larger randomized trials in the future could consolidate our present conclusions.

## Author contributions

**Conceptualization:** Chieh-Shou Su, Wen Lieng Lee.

**Data curation:** Ching-Hui Shen, Keng-Hao Chang, Chih-Hung Lai, Kuan-Ju Chen, Tzu-Hsiang Lin, Yu-Wei Chen.

**Investigation:** Ching-Hui Shen, Keng-Hao Chang, Chih-Hung Lai, Tsun-Jui Liu, Kuan-Ju Chen, Tzu-Hsiang Lin, Yu-Wei Chen.

**Methodology:** Wen Lieng Lee.

**Supervision:** Tsun-Jui Liu, Wen Lieng Lee.

**Writing – original draft:** Chieh-Shou Su, Ching-Hui Shen.

**Writing – review & editing:** Wen Lieng Lee.
